# Nitrate reductase is required for sclerotial development and virulence of *Sclerotinia sclerotiorum*

**DOI:** 10.3389/fpls.2023.1096831

**Published:** 2023-06-05

**Authors:** Junjun Wei, Chuanchun Yao, Zonghe Zhu, Zhimou Gao, Guogen Yang, Yuemin Pan

**Affiliations:** ^1^ Anhui Province Key Laboratory of Integrated Pest Management on Crops, Key Laboratory of Biology and Sustainable Management of Plant Diseases and Pests of Anhui Higher Education Institutes, School of Plant Protection, Anhui Agricultural University, Hefei, China; ^2^ College of Agronomy, Anhui Agricultural University, Hefei, China

**Keywords:** *Sclerotinia sclerotiorum*, nitrate reductase, SsNR, sclerotial development, infection cushion, virulence

## Abstract

*Sclerotinia sclerotiorum*, the causal agent of Sclerotinia stem rot (SSR) on more than 450 plant species, is a notorious fungal pathogen. Nitrate reductase (NR) is required for nitrate assimilation that mediates the reduction of nitrate to nitrite and is the major enzymatic source for NO production in fungi. To explore the possible effects of nitrate reductase SsNR on the development, stress response, and virulence of *S. sclerotiorum*, RNA interference (RNAi) of *SsNR* was performed. The results showed that *SsNR*-silenced mutants showed abnormity in mycelia growth, sclerotia formation, infection cushion formation, reduced virulence on rapeseed and soybean with decreased oxalic acid production. Furthermore *SsNR*-silenced mutants are more sensitive to abiotic stresses such as Congo Red, SDS, H_2_O_2_, and NaCl. Importantly, the expression levels of pathogenicity-related genes *SsGgt1*, *SsSac1*, and *SsSmk3* are down-regulated in *SsNR*-silenced mutants, while *SsCyp* is up-regulated. In summary, phenotypic changes in the gene silenced mutants indicate that SsNR plays important roles in the mycelia growth, sclerotia development, stress response and fungal virulence of *S. sclerotiorum*.

## Introduction

*Sclerotinia sclerotiorum* (Lib.) de Bary is a necrotrophic pathogen that causes white mold disease on many crops, including rapeseed, soybean, sunflower, peanut, and other economically important crops (Bolton et al., 2006). *Sclerotinia* stem rot (SSR) is one of the most important diseases on rapeseed in China, which causes significant yield losses and economic damage. SSR accounts for an estimated 10–30% of yield losses and may reach 80% in certain years. This fungus produces black sclerotia to overcome cold winters and survival in the field for many years (Adams and Ayers, 1979). When the average temperature is cool (8–14°C) during the flowering period of rapeseed, the prolonged humid or wet conditions are conducive to carpogenic germination of sclerotia-producing apothecia to release ascospores for infection and favor disease development. Under the new policy guidelines and the increasingly negative effects of traditional chemical control, elucidating the molecular mechanism of pathogenesis of *S. sclerotiorum* may provide important information for the effective control SSR of oilseed.

Currently, most studies mainly focus on the mechanism of action of pathogenic factors, such as hydrolase and oxalic acid (OA) secreted by *S. sclerotiorum* during infection. Cutinases secreted by *S. sclerotiorum* degrade the cuticle of host plants, promoting penetrating the epidermis cells. SsCut, a cutinase produced by *S. sclerotiorum*, could cause plant cell necrosis and induce host plant resistance (Zhang et al., 2014). Many plant cell wall degradation enzymes (PCWDEs) and proteases, including cellulases, hemicellulose, pectinases, xylanases, and aspartyl protease secreted by *S. sclerotiorum* to degrade host cell wall, and pectinases have received attention, especially polygalacturonases (PGs).

*S. sclerotiorum* has 183 PCWDEs (including lignase), among which 33 are pectin degradation enzymes (Amselem et al., 2011; Lyu et al., 2015; Seifbarghi et al., 2017). In addition, SsXyl1 (endo-onosine-1, 4-xylanase) is involved in the pathogenesis (Yu et al., 2016). Aspartate protease SsAp1 was significantly expressed during the early stage of infection on *Brassica napus* and *Phaseolus vulgaris* (Oliveira et al., 2015; Seifbarghi et al., 2017). OA plays an important role in the process of infecting host plants; the oxaloacetic acid hydrolase (OAH1, EC 3.7.1.1) deletion mutant, although able to infect the host plants, only causes smaller necrotic leaf lesions (Liang et al., 2015b; Xu et al., 2015).

Besides, many genes also participate in the pathogenesis of *S. sclerotiorum*, such as NADPH oxidase (SsNox1 & SsNox2) related to ROS production and the accumulation of oxalic acid (Kim et al., 2011). *Ss-Ggt1* (γ-glutamyl transpeptidase) is involved in the formation of infection cushions (Li et al., 2012), Cu/Zn superoxide dismutase SsSOD1 deficient resulting in significantly decreased virulence (Veluchamy et al., 2012; Xu and Chen, 2013). Forkhead-box transcription factor SsFKH1 regulates the development of the infection cushion, and silenced mutants produce small disease spots on tomatoes (Fan et al., 2017; Cong et al., 2022). However, elicitor SsPemG1 negatively regulated the pathogenicity of *S. sclerotiorum* (Pan et al., 2015). Moreover, secreted proteins play important roles in the penetration and regulation of plant immunity response, including SsCm1, *ssv263*, SSITL, Ss-Caf1, SsCVNH, SsSSVP1, Ss-Rhs1, SsCP1, and SsCut1 (Djamei et al., 2011; Liang et al., 2013; Zhu et al., 2013; Xiao et al., 2014; Lyu et al., 2015; Lyu et al., 2016a; Yu et al., 2017; Pan et al., 2018; Yang et al., 2018; Gong et al., 2022). Furthermore, SsSSVP1 and SsCP1 have been identified as effectors and target host QCR8 and PR1, respectively (Lyu et al., 2016a; Yang et al., 2018).

Nitrogen metabolism in fungi is a strictly regulated process that allows fungi to have the ability to utilize other nitrogen sources when the desired substrate is insufficient (Bolton and Thomma, 2008). Nitrate reductase (NR) is required for nitrate assimilation in fungi that mediates the reduction of nitrate to nitrite and have been confirmed in *Aspergillus nidulans*, *Neurospora crassa*, and *Aspergillus fumigatus* (Johnstone et al., 1990; Okamoto et al., 1991; Amaar and Moore, 1998). NR is widely distributed in ectomycorrhizal (ECM) fungi to utilize nitrate as an N source even though the nitrate supply is very low (Nygren et al., 2008). While NR-silenced in mycorrhizal fungus *Laccaria bicolor* resulted in the inhibition of symbiosis with *Populus* (Kemppainen et al., 2009). The nitrate reductase (NR) gene (niaD) is required for nitric oxide (NO) production in *A. nidulans* during conidiation (Marcos et al., 2016; Franco-Cano et al., 2021), while nitrate reductase NIA1 is essential for nitrate assimilation and dispensable for pathogenicity in *Magnaporthe oryzae* (Samalova et al., 2013).

Here, we characterized an NR gene in *S. sclerotiorum* and applied RNAi technology to reveal the function of SsNR in *S. sclerotiorum*. We show that SsNR is associated with mycelium growth and pathogenesis and that SsNR knockdown results in defective infection cushion formation.

## Materials and methods

### Fungal strains and culture conditions

The *Sclerotinia sclerotiorum* wild-type strain FXGD2, preserved at the Fungus Laboratory of Anhui Agricultural University, was cultured on PDA. *SsNR*-silenced mutants were cultured on PDA containing 180 μg/mL hygromycin B at 25°C in the dark. *S. sclerotiorum* strains were cultured on PDA at 25°C for 3–5 days in the dark, and mycelium was collected to extract genomic DNA and RNA. The extraction procedure of total RNA of *S. sclerotiorum* was based on the instruction of E.Z.N.A.TM Total RNA Kit I from (Omega Bio-Tek, Atlanta, USA), and the extracted RNA was treated with RNAse-free DNAse I (TaKaRa Biotechnology, Dalian, China).

### Gene cloning and bioinformatics analysis

BLASTp searches were performed from the *S. sclerotiorum* genome database at National Center for Biotechnology Information (NCBI). A homolog of nitrate reductase (NR) was retrieved from the *S. sclerotiorum* genome and was named SsNR (SS1G_01885). The ORF of SsNR was amplified using primer *SsNR*-F/*SsNR*-R (**Table S1**), then cloned into pMD19-T for sequencing. ClustalX 2.1 (Larkin et al., 2007) was used for multiple sequence alignment of the SsNR and its homologs, and the phylogenetic tree was reconstructed by MEGA11 using maximum likelihood method (Tamura et al., 2021). The conserved motifs of the nitrate reductase family were analyzed at a web resource SMART (Letunic et al., 2021).

### Nucleotide acid extraction and quantitative reverse transcription-PCR

Total RNA was extracted from *S. sclerotiorum* strains according to the instruction of E.Z.N.A.TM Total RNA Kit I (Omega Bio-Tek, USA), and contaminant genomic DNA was removed with RNAse-free DNAse I (TaKaRa, Dalian, China). qRT-PCR was performed using SYBR Green RT-PCR Kit (TaKaRa, Dalian, China) to analyze the expression level of *SsNR* during mycelium growth and sclerotia formation and to confirm the *SsNR*-silenced mutants with house-keeping gene *β-tubulin* (*SsTub*, SS1G_04652) as reference gene. For gene expression of pathogenicity-related genes *SsGgt1* (SS1G_14127), *SsSmk3* (SS1G_05445), *SsSac1* (SS1G_07715) and *SsCyp* (SS1G_06284) in *SsNR*-silenced mutants, total RNA was extracted from strains and transcribed into cDNA for qRT-PCR using CFX96 thermal cycler (Bio-Rad, CA, USA). For each gene, qRT-PCR was repeated at least twice with three biological replicates.

### Construction of RNAi vector and transformation of *S. sclerotiorum*

pSilent-1, which carries a hygromycin resistance cassette, was used for hairpin RNA expression of *SsNR* (Nakayashiki et al., 2005). Two fragments amplified from cDNA using *SsNR-H-L*/SsNR*-X-R* and *SsNR-K-L*/*SsNR-S-R* (**Table S1**) were ligated into pSilent-1 respectively, to generate pSilent-*SsNR*. Plasmids of pSilent-*SsNR* were transferred into protoplasts of FXGD2 with polyethylene glycol (PEG)-mediated transformation (Rollins, 2003). Hygromycin-resistant colonies on RM were transferred to new PDA plates containing 45 μg/mL hygromycin B, and transformants of pSilent-1 were used as control. To confirm all putative transformants, Primers Hyg-F and Hyg-R were used to amplify the partial sequence of the hygromycin resistance gene.

### Mycelia growth, sclerotia, and formation of infection cushions

For mycelia growth and sclerotia formation, wild-type, *SsNR*-silenced transformants and mock strains were inoculated on PDA plates at 25°C for 2 d or 15 d. For infection cushion formation, agar plugs of all strains were inoculated onto the surfaces of glass slides and incubated at 25°C with 100% relative humidity, the number of infection cushion were counted at 24 h. All experiments were repeated three times independently.

### Determination of stress tolerance

For stress tolerance assay, fresh mycelium plugs of wild-type, *SsNR*-silenced transformants and mock strains were cultured on MM medium containing Congo Red (2 mg/mL), SDS (0.01%), sorbitol (1.2 M), NaCl (1 M), and H_2_O_2_ (4 mM), respectively. Colony diameters were measured every 12 h, and sclerotia formation was observed at 7 days. Experiments were repeated three times with three biological replicates.

### Quantification of oxalic acid

Strains were cultured on MM medium containing 0.01% bromophenol blue for qualitative analysis of oxalic acid production. For quantification of oxalic acid, mycelium plugs of different strains were cultured in 100 mL YSPU medium at 25°C for 5 days, and the content of oxalic acid secreted in YSPU was measured by KMnO_4_ titration method (Baker, 1952). This experiment was repeated three times.

### Pathogenicity assay

Rapeseed and soybean plants were used for the pathogenicity assay of *S. slcerotiorum* wild-type, *SsNR*-silenced and mock strains. Detached leaves were inoculated with mycelia plugs (Φ=5 mm) from the margins of actively growing colonies on PDA in an incubator at 18°C and 100% relative humidity. Disease severity was calculated by lesion percentage of leaf and lesion size. These experiments were repeated three times, and each replicate was performed with three leaves.

### Statistical analysis

All experiments were repeated three times. Microsoft Office 365 and SPSS v22.0 were used for statistical analysis. Statistical analysis was performed using Student’s t-test and one-way analysis of variance (ANOVA).

## Results

### SsNR is a putative nitrate reductase

**Figure 1:**
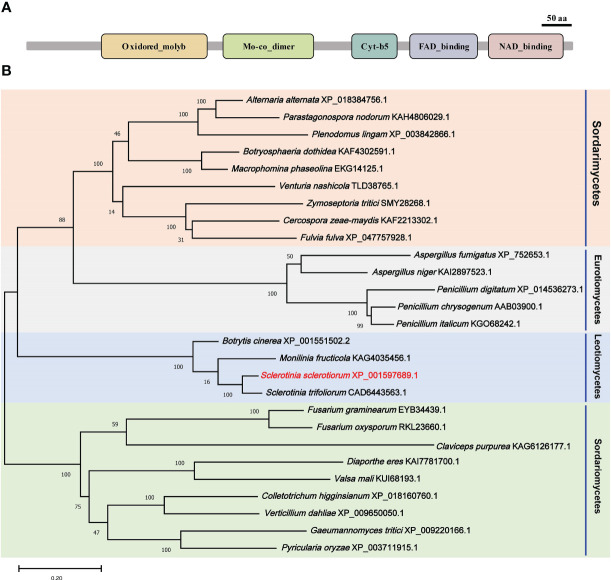
Functional domain identification and phylogenetic tree reconstruction. **(A)** Functional domains of SsNR were identified by searching SMART containing an Oxidored_molyb, Mo-co_dimer, Cyt-b5 domain, FAD_binding_6 domain, and NAD_binding_1. **(B)** Phylogenetic tree reconstructed using the maximum likelihood (ML) method with 1,000 bootstraps.

A nitrate reductase SsNR was identified from the *S. scelrotiorum* genome, and the ORF of *SsNR* is 2733 bp in length, encoding 910 amino acids. SsNR, like other nitrate reductases, has five functional domains: Oxidored_molyb structural domain (122-298 aa), Mo-co_dimer structural domain (326-475 aa), Cyt-b5 structural domain (541- 613 aa), FAD_binding_6 structural domain (645-751 aa) and NAD_binding_1 structural domain (771-895 aa) (**Figure 1A**). Phylogenetic analysis indicated that orthologs of SsNR are widely distributed in fungi and plant (**Figure 1B**).

### SsNR is essential for mycelium growth and sclerotial development

**Figure 2:**
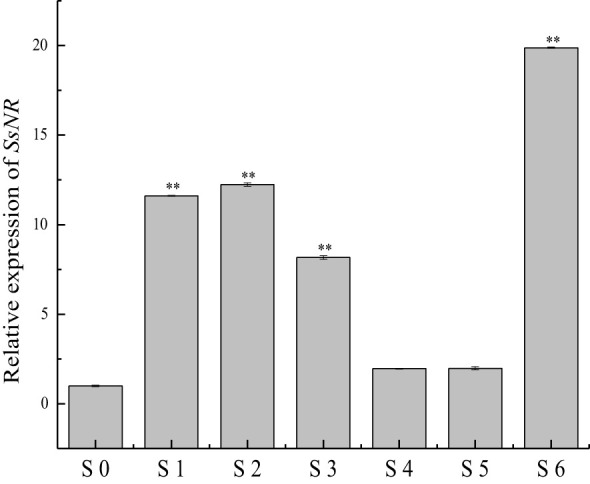
qRT-PCR analysis of the expression of *SsNR* in different stages of *S. sclerotiorum*. S0 = the hyphal stage of *S. sclerotiorum*; S1 = the initiation stage of sclerotial development; S2 = condensation stage; S3 = enlargement stage; S4 = consolidation stage; S5 = pigmentation stage; S6 = maturation stage. The expression level of SsNR cDNA measured by qRT-PCR was standardized with the housekeeping gene *S. sclerotiorum β-tubulin*. The abundance of cDNA from S0 samples was assigned a value of 1. Bars indicate standard error. Statistical significance is indicated: **, *P *<* *0.01.

**Figure 3:**
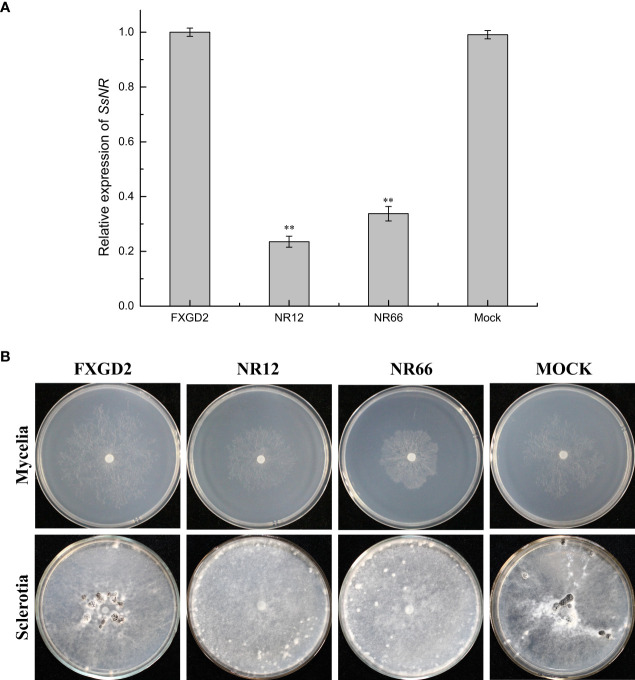
*SsNR*-silenced impaired hyphae growth and sclerotia development. **(A)** qRT-PCR analysis of the expression of *SsNR* in WT, *SsNR*-silenced mutants and mock. Statistical significance is indicated: **, *P *<* *0.01. **(B)** Colony morphology of WT, *SsNR*-silenced mutants and mock cultured on PDA at 25°C. Photos were taken at 2 dpi, and the sclerotia of WT*, SsNR*-silenced mutants and mock cultured on PDA at 25°C. Photos were taken at 10 dpi.

qRT-PCR was used for *SsNR* expression analysis during hyphal growth and six stages of sclerotial formation: (S1) initiation, (S2) condensation, (S3) enlargement, (S4) consolidation, (S5) pigmentation, and (S6) maturation (Li and Rollins, 2009). Our qRT-PCR results showed that *SsNR* is highly expressed from S1 to S6. The transcript level increased by 1060%, 1124%, 717%, 96%, 98%, and 1888%, respectively, indicating that SsNR might be involved in the sclerotial development of *S. sclerotiorum* (**Figure 2**). We performed PEG-mediated transformation. We obtained 82 transformants that could grow on PDA-containing hygromycin. *SsNR*-silenced mutants were confirmed by qRT-PCR, and two mutants (NR12 and NR66) were chosen for analysis in which the expression of *SsNR* decreased by 76.5% and 66.3%, respectively (**Figure 3A**). *SsNR*-silenced mutants exhibited slower mycelial growth compared to wild-type and mock strain (**Figure 3B**). The wild-type and mock strain produced sclerotia in 6 days post-inoculation, but *SsNR*-silenced mutants produced few sclerotia or did not form scerotia, indicating that SsNR was involved in mycelium growth and sclerotial development (**Figure 3B**).

### SsNR is involved in response to Congo red, SDS, NaCl, and H_2_O_2_

**Figure 4:**
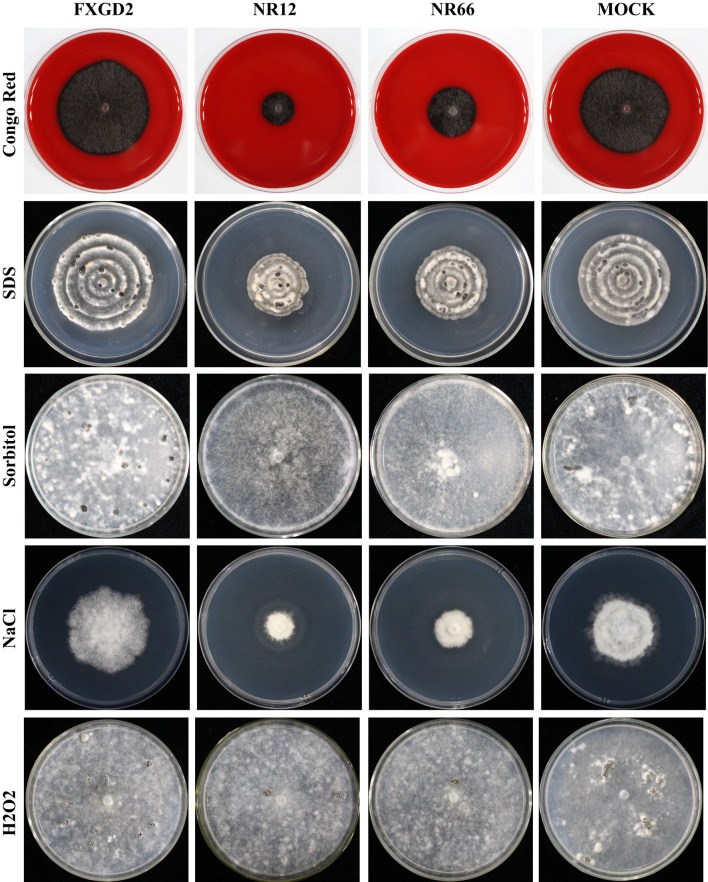
Tolerance of WT, *SsNR*-silenced mutants and mock to different chemicals. The phenotype of WT, *SsNR*-silenced mutants and mock grown on MM supplemented with 2 mg/mL Congo Red, 0.01% SDS, 1.2 M Sorbitol and 1 M NaCl and 4 mM H_2_O_2_. Photos were taken at 1 dpi, 5 dpi, 7 dpi, 2 dpi, and 7 dpi, respectively.

To check the potential role of nitrate reductase in the cell wall and membrane integrity of *S. sclerotiorum*, *SsNR*-silenced mutants were cultured on MM containing Congo Red (2 mg/mL) and SDS (0.01%), respectively. Under Congo Red treatment, the colony diameter of *SsNR*-silenced mutants is about 1.85 and 2.72 cm, much smaller than the wild-type (4.93 cm) and mock strain (4.67 cm). Similarly, *SsNR*-silenced mutants grew much slower than wild-type and mock strain on an SDS-amended medium. The colony diameters of wild-type, mock strain and *SsNR*-silenced mutants (NR12 and NR66) were 5.75, 5.45, 3.3, and 4.1, respectively (**Figure 4**). These results showed that SsNR is required for cell wall integrity.

We also detect the response of different strains to other stress -including chemicals, such as sorbitol, NaCl, and H_2_O_2_. The results showed that colony morphology of *SsNR*-silenced mutants did not change on MM containing sorbitol and that the mycelium growth and sclerotial development were not affected, indicating that SsNR is not associated with osmotic pressure (**Figure 4**). NaCl at 1 M could significantly inhibit mycelium growth of *SsNR*-silenced mutants compared to wild-type and mock. Likewise, in *SsNR*-silenced mutants grown on MM containing H_2_O_2_, the diameter of the colony was much smaller (3.1 and 4.6 cm) compared to wild-type (6.0 cm), indicating that SsNR has the function of tolerance to H_2_O_2_ (**Figure 4**).

### SsNR is associated with hyphal branching and infection cushion development

**Figure 5:**
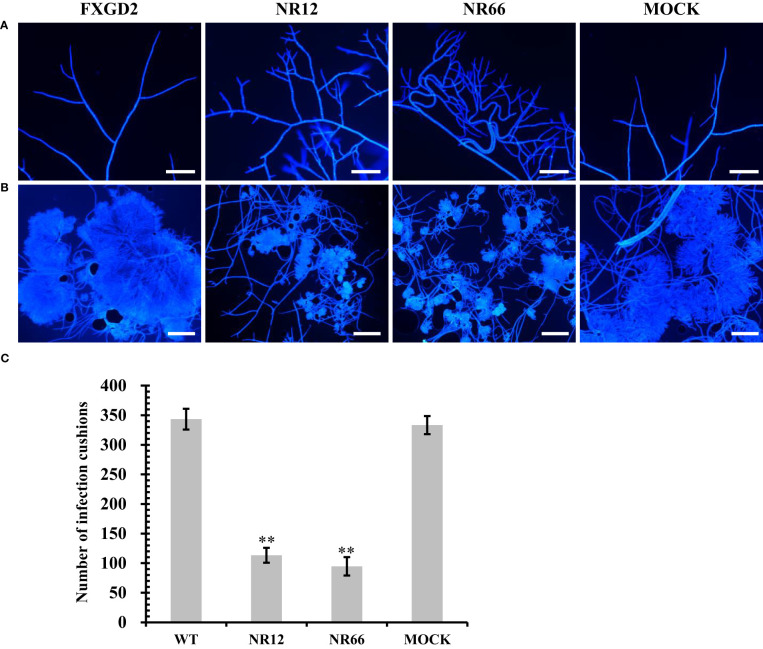
Mycelial growth and infection cushion formation of *SsNR*-silenced mutants. **(A)** Hyphal branching and tips stained of all strains were observed under a light microscope. **(B)** Infection cushions stained of all strains were observed under a light microscope. **(C)** Number of infection cushion produced by all strains. Bar = 100 μm, hyphae and infection cushions were by Calcofluor white (CFW) (10 μg/mL). Statistical significance is indicated: **, P < 0.01.

For *SsNR*-silenced mutants that exhibited abnormal colony morphology, we detected the hyphal tip under a microscope to determine whether SsNR affects hyphal growth (**Figure 5A**). The result showed that the hyphal branching of *SsNR*-silenced mutants increased in the number of tips, and the hyphal diaphragm was shortened compared to the wild-type. The infection cushions played important roles during penetration of *S. sclerotiorum*; we detected the number of infection cushions produced by different strains on hydrophobic surfaces that the *SsNR*-silenced mutants produce less and smaller infection cushions compared to WT (**Figures 5B, C**). These results indicated that SsNR plays an important role in infection cushions formation.

### SsNR is required for virulence and oxalic acid accumulation

**Figure 6:**
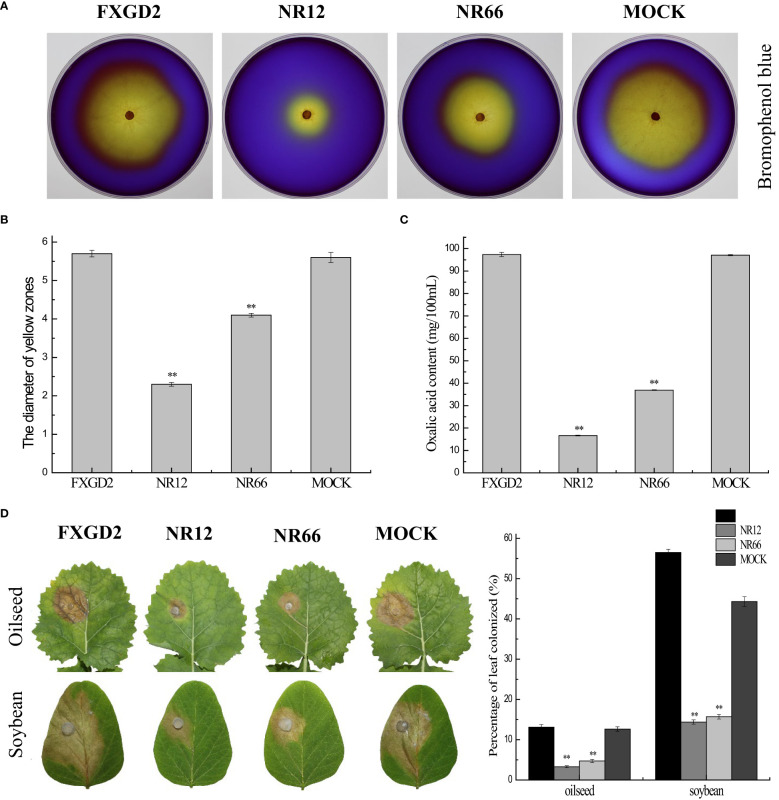
*SsNR* is required for virulence and accumulation of oxalic acid in *S. sclerotiorum.* **(A)** Morphology of WT, *SsNR*-silenced mutants and mock grown on MM supplemented with 0.01% bromophenol blue. Photos were taken at 2 dpi. **(B)** The diameters of the yellow zone produced by WT, *SsNR*-silenced mutants and mock (2 dpi). **(C)** Oxalic acid concentration of WT, *SsNR*-silenced mutants and mock cultured in liquid medium 5 days. **(D)** The percentages of lesion area produced by WT, *SsNR*-silenced mutants and mock-inoculated on detached leaves of oilseed rape and soybean (48 hpi). Statistical significance is indicated: **, P < 0.01.

For SsNR is associated with mycelium growth, we also performed a pathogenicity assay on detached leaves of rapeseed and soybean plants. The results showed that SsNR-silenced mutants could produce lesions on leaves of both rapeseed and soybean (12.64%~13.12% and 44.36%~56.50%), but the lesion size of mutants was much smaller (3.28%~4.73% and 14.39%~15.72%) than wild-type and mock strains, indicating that SsNR is required for infection of *S. sclerotiorum* (**Figure 6**).

Oxalic acid plays an important role in pathogenicity during infection. All strains were cultured on a medium containing bromophenol to determine the OA in wild-type and SsNR-silenced mutants. The diameters of the yellow zone produced by silenced transformants NR12 and NR66 were about 2.3 and 4.1 cm, respectively, which were significantly smaller than those of the wild-type (5.7 cm) and mock control (5.6 cm) (**Figure 6**). Also, the concentration OA of all strains were determined by KmnO_4_ titration, results showed that OA concentration of NR12 and NR66 were 16.65 mg and 36.9 mg in 100 ml medium, respectively, while those of wild-type and mock strain were 97.35 mg and 97.05 mg, respectively (**Figure 6**). These results showed that SsNR is important for OA production.

### Regulation of the expression of pathogenicity-related genes of *S. sclerotiorum*

**Figure 7:**
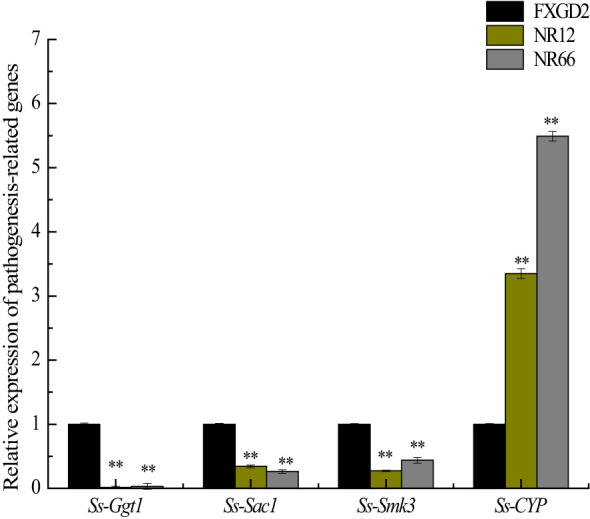
The expression of *SsGgt1*, *SsSac1*, *SsSmk3*, and *SsCYP* in different strains using *β-tubulin* as a reference gene. Statistical significance is indicated: **, P < 0.01.

To investigate whether *SsNR*-silencing affects the transcriptional expression levels of other pathogenicity-related genes, *Ggt1*, *Sac1*, *Smk3*, and *CYP*, were analyzed by qRT-PCR. The results showed that the expression levels of *Ggt1*, *Sac1*, and *Smk3* in *SsNR*-silenced mutants were decreased by 96%~98%, 65%~74%, and 56%~72%, respectively. At the same time, the expression level of *CYP* was increased by 230%~440% (**Figure 7**).

## Discussion

Nitrate reductase is an enzyme that catalyzes the reduction of nitrate to nitrite and is the major enzymatic source for NO production in fungi (Cánovas et al., 2016). The nitrate reductase SsNR of *S. sclerotiorum* was studied using sequence analysis and RNAi technology which could be a good approach for gene function analysis in this fungus (Lyu et al., 2016a; Rana et al., 2021). *SsNR*-silenced mutants exhibited altered phenotypes, including hyphal growth and sclerotia development. We performed a pathogenicity assay on detached leaves of rapeseed and soybean plants; although the *SsNR*-silenced mutants could penetrate the host cell, they produced smaller lesions than WT.

*SsNR*-silenced mutants exhibited increased hyphal branching tips and shortened hyphal diaphragm, and diminished sclerotia development (**Figure 3**). These results indicated that mycelium growth might be related to sclerotia development, and these phenomena are also observed in knockout or knock down mutants of other genes, such as *Sop1*, *Ss-Sl2*, *SsFkh1*, *SCD1*, and *THR1* (Yu et al., 2012; Lyu et al., 2016b; Fan et al., 2017; Yue et al., 2018). Also, endogenous small RNAs may regulate genes controlling sclerotial development (Xia et al., 2020). Nitrate reductase *SsNR*-silenced mutants may affect nitrogen metabolism and protein synthesis, which might cause abnormal hyphal growth and sclerotia formation. An infection cushion (or compound appressorium) is required for infection, which is the primary means of infection initiation by *S. sclerotiorum* to breach the cuticle layer of the host epidermal cell (Huang et al., 2008). *SsNR*-silenced mutants produced less infection cushion on parafilm. SsNR could regulate the development of infection cushion accomplished with decreased virulence (**Figure 5**). Several genes have been reported in regulating infection cushion development, such as *Sac1*, *Ss-caf1*, *Ss-oah1*, *Ss-odc2*, and *Smk3* (Jurick and Rollins, 2007; Li et al., 2012; Xiao et al., 2014; Liang et al., 2015a; Liang et al., 2015b; Bashi et al., 2016), meanwhile *Ggt1*, *Sac1*, and *Smk3* are downregulated in *SsNR*-silenced mutants, indicating that SsNR is involved in the expression of infection cushion development-related genes in fine-tuning the infection cushion formation process during penetration of *S. sclerotiorum*.

OA is important for infection of *S. sclerotiorum* primarily in acidifying the microenvironment in infection (Xu et al., 2018), in addition to other roles, including chelation with Ca^2+^, regulation stomatal closure, inhibition of reactive oxygen species (ROS) bursting, promotion of apoptosis, and repression autophagy of plant cell (Rollins and Dickman, 2001; Guimaraes and Stotz, 2004; Williams et al., 2011; Kabbage et al., 2013; Uloth et al., 2015; Derbyshire et al., 2022). OA production of *SsNR*-silenced mutants decreased compared to WT; we proposed that SsNR may play an important role in OA accumulation, implying that SsNR regulated the pathogenicity of *S. sclerotiorum* through oxalic acid secretion. For decreased OA concentration, *SsNR*-silenced mutants produced fewer infection cushions and impaired function of OA during plant-*Sclerotinia* interaction, causing debilitation in virulence. Moreover, cell wall integrity (CWI) is required in fungi to adapt to perturbing conditions, including osmotic pressure, heat, oxidative stress, and antifungals (Dichtl et al., 2016). *SsNR*-silenced mutants were more sensitive to Congo Red, SDS, and NaCl than the wild type, indicating impaired CWI. Reactive oxygen species (ROS) played important roles in plant immunity (Qi et al., 2017), while *SsNR*-silenced mutants showed more sensitivity to H_2_O_2_ compared to WT resulting in decreased virulence in plants (soybean and rapeseed). Similar results were obtained in the tea leaf spot which is caused by *Didymella segeticola*, the antimicrobial kasugamycin inhibits the pathogen by binding to NR, disturbing fungal metabolism with changes in hyphal growth and development (Jiang et al., 2022).

## Conclusion

SsNR is essential for normal mycelium growth, sclerotia development, and virulence by regulating OA production and expression of pathogenesis-related genes involved in the infection of *S. sclerotiorum*.

## Data availability statement

The original contributions presented in the study are included in the article/**Supplementary Material**. Further inquiries can be directed to the corresponding authors.

## Author contributions

ZG, GY, and YP designed the research. JW and CY conducted the experiments and data analysis. JW, CY, ZZ, ZG, GY, and YP wrote and revised the manuscript. All authors scrutinized and corrected the manuscript. All authors contributed to the article and approved the submitted version.
