# Supplementary material for: Nitrate reductase is required for sclerotial development and virulence of Sclerotinia sclerotiorum
Source: Front Plant Sci. 2023 Jun 5;14:1096831. doi: 10.3389/fpls.2023.1096831 (PMC10277653; doi:10.3389/fpls.2023.1096831)
Supplement: Supplementary file 1 [file Table_1.docx]

Supplementary Material

# Supplementary Tables

Table 1 Primers listed in article.

| Primers | Primer sequence (5’-3’) | ACCESSION | Restriction enzyme cutting sites |
| --- | --- | --- | --- |
| *SsNR*-F | CCG**CTCGAG**ATGGCCAGTGCGAATTATATAGATGGGC | XM_001597639.1 | *XhoI* |
| *SsNR*-R | G**GCATGC**CTAAAAGAACAATAAATCTTGATCCCTCCACTCC |  | *SphI* |
| *SsNR*-H-F | CCC**CTCGAG**GGAAAGAGGAGGAAGGTACGGAGATT |  | *Xho I* |
| *SsNR*-X-R | CCG**AAGCTT**AAACCAGCTGTTCCCCATCCGCATAC |  | *Hind III* |
| *SsNR*-K-F | CGG**GGTACC**GGAAAGAGGAGGAAGGTACGGAGATT |  | *Kpn I* |
| *SsNR*-S-R | ACAT**GCATGC**AAACCAGCTGTTCCCCATCCGCATAC |  | *Sph I* |
| RT-*SsNR*-F | GGTCGCAGTGTGAAATGGTTG |  |  |
| RT-*SsNR*-R | GCGTATCTCTCATCCGTCCAC |  |  |
| RT-*β-tubulin*-F | TTGGATTTGCTCCTTTGACCAG | XM_001594794.1 |  |
| RT-*β-tubulin*-R | AGCGGCCATCATGTTCTTAGG |  |  |
| RT-*Fusion*-F | ACCCCAAAGAAGATCAAGCAC | XM_001587745 |  |
| RT-*Fusion*-R | CCACAGTATTGACGGTCGTG |  |  |
| RT-*Cyp*-F | ACTTCCGTGCTCTTTGCAC | NW_001820829 |  |
| RT-*Cyp*-R | GTGCTTTCGCTCGAAGTTCT |  |  |
| RT-*Smk3*-F | GGAAACCGATATGCATCGTGT | NW_001820816 |  |
| RT-*Smk3*-R | ACCTTCAAATCGCAGTTGGC |  |  |
| RT-*SsSac1*-F | GTTGGAGCATGCCGAACTAT | XM_001591040.1 |  |
| RT-*SsSac1*-R | CGATCAAAGCAGTCATGTTGC |  |  |
| RT-*Ggt1*-F | CGGCACCGTCTACATTTCTC | XM_001584794.1 |  |
| RT-*Ggt1*-R | TCTGAAGAAAGCCGTGGTCT |  |  |
| *hyg*-F | ATGAAAAAGCCTGAACTCACCGCGAC | AB303070  REGION: 2593..3618 | |
| *hyg*-R | CTATTCCTTTGCCCTCGGACGAGT |  |  |

a. Bold letters show restriction enzyme cutting sites.
